# A Case of Multicentric Carcinoid in a Patient with Psoriatic Spondyloarthropathy

**DOI:** 10.1155/2015/179696

**Published:** 2015-02-23

**Authors:** Nabil George, Archana Depala, Laith Al Sweedan, Kuntal Chakravarty

**Affiliations:** ^1^North Central Thames Foundation School, London NW3 2P, UK; ^2^George Eliot Hospital NHS Trust, Nuneaton CV10 7DJ, UK; ^3^Royal Free Hospital NHS Foundation Trust, London NW3 2QG, UK; ^4^Addenbrooke's Hospital, Cambridge CB2 0QQ, UK

## Abstract

We describe the first case of a patient presenting with multicentric carcinoid occurring in the lung and subsequently in the rectum, with chronic psoriatic arthritis. Although reports have been published regarding carcinoid syndrome occurring alongside rheumatoid arthritis, no reports have been made on such a case. Initial presentation of carcinoid syndrome in this patient was insidious and atypical with few symptoms, including shortness of breath and long standing abdominal bloating. Several years later a sudden change in bowel habit prompted a colonoscopy with biopsy that revealed a carcinoid rectal polyp. The case we report describes a rare presentation of carcinoid syndrome in chronic psoriatic arthropathy.

## 1. Introduction

Psoriatic srthritis (PsA) is a chronic inflammatory disease of the synovial joints associated with skin psoriasis [1]. Carcinoid tumours, on the other hand, are the commonest endocrine neoplasms of the gut [2], mainly found in the small bowel, derived from neuroendocrine enterochromaffin (and argentaffin) cells [3]. The high incidence of carcinoid in the bronchopulmonary tract (25.3% of all carcinoids) relates to its embryonic gut-origin [4]. We report a case of a 54-year-old Caucasian female with an 11-year history of psoriasis associated with enthesopathy and multicentric neuroendocrine tumour, hitherto unreported in the literature. The diagnosis of psoriatic arthritis was confirmed in 2009 and she was treated with conventional drugs and UVB therapy, with moderate clinical response and subsequent surgical therapy for the tumours.

## 2. Case

**Figure 1:**
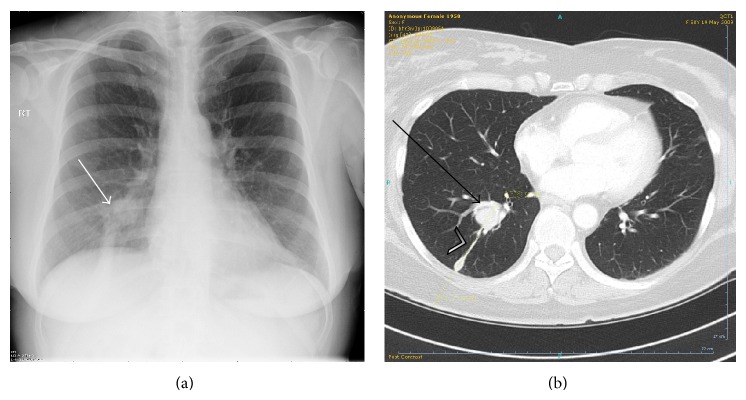
(a) Chest X-ray showed a 2 cm round opacity (white arrow) over the lower end of the right hilum with an inferiorly extending band shadow. (b) Transverse section chest contrast-enhanced computed tomography confirmed a 26 mm by 23 mm irregularly round lesion (black arrow) located at the bifurcation of the posterior segmental division of the lower lobe bronchus. There is further extension posteroinferiorly of the irregular soft tissue (arrowhead) contiguous from the lesion towards the costophrenic angle and diaphragm with pleural thickening.

We present a case of a 54-year-old Caucasian lady, who three months following the diagnosis of PsA, presented with persistent cough and worsening dyspnoea at her outpatient clinic appointment. On examination, the patient had reduced chest expansion on the right side and a dull percussion note at the right lower zone. There was no clubbing and no lymphadenopathy. Urgent chest X-rays showed a suspicious 2 cm lesion in the right lower zone. During this time, the patient was being worked up for specific disease-modifying antirheumatic drug (DMARD) therapy, which was halted due to the unusual chest symptoms and imaging abnormalities. CT-guided transthoracic biopsy confirmed a 26 × 23 mm soft tissue lesion located at the bifurcation of the lower lobe bronchus. No mediastinal lymphadenopathy was noted. Histological analysis of the biopsy tissue revealed a benign typical carcinoid tumour (Figure 1).

**Figure 2:**
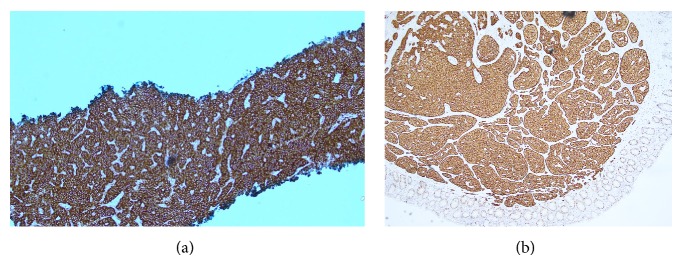
Biopsy specimens from lung (a) and rectal polyp (b). (a) Histological analysis of lung lesion revealed columnar cells with bland nuclei arranged in an insular pattern with infrequent mitoses. Staining for neuron-specific enolase, synaptophysin, and CD56 was highly positive, but negative for chromogranin A and S100, suggestive of typical carcinoid neoplasia. (b) Rectal polyp analysis showed well circumscribed growth of uniform polygonal cells with round nonmitotic nuclei and speckled chromatin with nested and acinar growth pattern; features indicative of a benign endocrine tumour. Histologically, the specimen from the rectum was not suggestive as being related to the lung lesion, hence the labelling of this case as “multicentric.”

Consequently, a right lower lobectomy was performed, with complete resection of the tumour. The patient was monitored without requiring any further treatment. Following the successful resection of the lesion, a 6-week trial of Methotrexate was commenced specifically for PsA but did not prove efficacious. She was then switched to Leflunomide with a better clinical response. Ten months after the lobectomy, the patient complained of new onset night sweats, weight loss, and flushing, albeit with a 2-year background of abdominal bloating. Two months later, she experienced a change in bowel habit. An urgent colonoscopy revealed a rectal polyp that was resected as per standard procedure. Benign typical carcinoid was confirmed histologically (Figure 2). An octreotide scan and 5-hydroxyindoleacetic acid (5HIAA) urine levels were negative following polypectomy.

She was diagnosed to have multicentric carcinoid syndrome (CS), in the background of PsA. This was a histopathological diagnosis and not based on clinical evidence of more than one site involvement. Interestingly, a small number of patients with CS develop symptoms of arthritis compared to the general population, the causes for which remain elusive [5]. Albeit, paraneoplastic syndromes such as CS can rarely present in patients with Rheumatoid Arthritis [6], where three cases have been reported in the literature. However, to our understanding none have been reported for patients with PsA [7]. This is likely to be incidental as there is no recognised relationship between PsA and CS.

## 3. Discussion

To our knowledge and extensive literature reviews, the diagnosis of multicentric CS in a patient with PsA has not previously been reported. There are also no reports of Leflunomide causing a clinically significant rise in the incidence of cancers as an adverse effect [8], only a theoretical potential risk for solid tumours [9]. However, 18 months after commencing Leflunomide, the patient experienced lower GI symptoms. Although diarrhoea is a recognised side effect of Leflunomide therapy, it is important to sufficiently investigate patients with PsA treated with DMARD that present with persistent symptoms related to the bowel or lung, as there could be a sinister underlying cause. This case highlights the unusual presentation of paraneoplastic syndromes, such as CS, in background of psoriasis related arthropathy. As clinicians, it is important to be aware of such atypical and insidious presentations in order to prevent them from being overlooked.

## Key Message

A case of PsA is described, complicated by carcinoid syndrome, as previously unreported in the literature.
